# German radiation oncology’s next generation: a web-based survey of young biologists, medical physicists, and physicians—from problems to solutions

**DOI:** 10.1007/s00066-024-02305-8

**Published:** 2024-10-22

**Authors:** Thomas Weissmann, Lisa Deloch, Maximilian Grohmann, Maike Trommer, Alexander Fabian, Felix Ehret, Sarah Stefanowicz, Alexander Rühle, Sebastian Lettmaier, Florian Putz, Maya Shariff, Simone Wegen, Johann Matschke, Elena Sperk, Annemarie Schröder

**Affiliations:** 1 Department of Radiation Oncology, Uniklinikum Erlangen, Friedrich-Alexander-Universität Erlangen-Nürnberg, Erlangen-Nürnberg, Germany; 2 Comprehensive Cancer Center Erlangen-EMN, Erlangen, Germany; 3 Young DEGRO Trial Group, Berlin, Germany; 4 Translational Radiobiology, Department of Radiation Oncology, Uniklinikum Erlangen, Friedrich-Alexander-Universität Erlangen-Nürnberg, Erlangen-Nürnberg, Germany; 5 Department of Radiotherapy and Radiation Oncology, University Medical Center Hamburg-Eppendorf, Martinistr. 52, 20246 Hamburg, Germany; 6 Department of Radiation Oncology, University of Cologne, Faculty of Medicine and University Hospital Cologne, Cologne, Germany; 7 Department of Radiation Oncology, Olivia Newton-John Cancer Wellness & Research Centre, Austin Health, Melbourne, VIC Australia; 8 Department of Radiation Oncology, University Hospital Schleswig-Holstein, 24105 Kiel, Germany; 9 Charité—Universitätsmedizin Berlin, Corporate Member of Freie Universität Berlin and Humboldt-Universität zu Berlin, Department of Radiation Oncology, Berlin, Germany; 10 Department of Radiation Oncology, Klinikum rechts der Isar, Technical University of Munich (TUM), Munich, Germany; 11 Department of Radiation Oncology, University of Freiburg—Medical Center, Freiburg, Germany; 12 Department of Radiation Oncology, University of Leipzig, Leipzig, Germany; 13 Department of Radiation Oncology, Cyberknife and Radiotherapy, Faculty of Medicine and University Hospital Cologne, Cologne, Germany; 14 Institute of Cell Biology (Cancer Research), University Hospital Essen, University of Duisburg-Essen, 45147 Essen, Germany; 15 German Cancer Consortium (DKTK) partner site Essen a partnership between DKFZ and University Hospital, Essen, Germany; 16 Mannheim Cancer Center, Universitätsmedizin Mannheim, Medizinische Fakultät Mannheim, Universität Heidelberg, Heidelberg, Germany; 17 Department of Radiotherapy and Radiation Oncology, University Medical Center Rostock, Suedring 75, 18059 Rostock, Germany; 18 Comprehensive Cancer Center MV, Rostock, Germany

**Keywords:** Radiation research, Radiation oncology, Translational research, Young workforce, Work conditions

## Abstract

**Background:**

Radiation science is of utmost significance not only due to its growing importance for clinical use, but also in everyday life such as in radiation protection questions. The expected increase in cancer incidence due to an aging population combined with technical advancements further implicates this importance and results in a higher need for sufficient highly educated and motivated personnel. Thus, factors preventing young scientists and medical personnel from entering or remaining in the field need to be identified.

**Methods:**

A web-based questionnaire with one general and three occupation-specific questionnaires for physicians, biologists, and medical physicists working in radiation oncology and research was developed and circulated for 6 weeks.

**Results:**

While the overall satisfaction of the 218 participants was quite high, there are some points that still need to be addressed in order to ensure a continuing supply of qualified personnel. Among these were economic pressure, work–life balance, work contracts, protected research time, and a demand for an improved curriculum.

**Conclusion:**

Mentoring programs, improved education, and strengthening the value of societies in radiation sciences as well as translational approaches and more flexible working arrangements might ensure a high-quality workforce and thus patient care in the future.

**Supplementary Information:**

The online version of this article (10.1007/s00066-024-02305-8) contains supplementary material, which is available to authorized users.

## Introduction

Radiation science is of the utmost significance not only due to its growing importance for clinical use, but also regarding safety issues related to environmental exposure to radiation, nuclear build outside of Germany, and decommissioning of existing power plants or possibly due to space travel [1]. Because of the expected increase in cancer incidence (2.5% annually) due to an aging population and because of faster improvements and prevention measures in cardiovascular treatment, cancer is predicted to overtake cardiovascular disease as the leading cause of death. This further marks the growing importance of radiation in clinical cancer treatment and diagnostics [2, 3]. Surveys of the European Union show that 45% of all cancer patients are cured. Of these 45%, 22% undergo surgery alone, 12% radiotherapy alone, 6% a combination of surgery and radiotherapy, and 5% multimodal treatment combining surgery, radiotherapy, and chemotherapy [4, 5]. Moreover, radiotherapy is often implemented in palliative care to reduce symptomatic burden in advanced disease [6, 7]. Overall, these developments clearly demonstrate that ionizing radiation should not only be considered a risk factor for harmful effects in the public [8] but also as a crucial factor for cancer treatment benefitting the public overall. In recent years, the field of radiation oncology has seen significant advances in therapeutic techniques, with new insights into radiation biology as well as advanced treatment planning algorithms, together contributing to improved quality of life and prolonged overall survival in patients with cancer [9, 10].

For application of advanced radiation therapy in the clinical context, various professional groups are needed, all of which make an important contribution to the overall treatment concept and success [11]. Overall, technicians make up the biggest cohort of staff in radiation oncology and are of utmost importance; the following survey, however, focusses on the academic workforce. Here, physicians represent the largest cohort. The other academic professional groups include radiation biologists and medical physicists [2, 11]. All of these persons are of utmost importance not only for ensuring high-quality treatment and safety, but also to achieve the best possible clinical outcomes for patients and to pave the way to future scientific progress. The need for improved translational and multidisciplinary work has also been addressed by the “Vision 2030 for radiotherapy & radiation oncology in Germany” [12]. To achieve this goal, the different specialties have different focus points: physicians usually have a primarily clinical focus on patient care. Medical physicists are more involved in technical treatment planning, accurate dosimetry, safe and effective delivery of radiation, and the implementation of new technologies [13]. Biologists, on the other hand, usually focus on scientific questions, often work in laboratories outside of patient care, and are often challenged by the existing structures of the scientific world such as reduced and thus inadequate funding and/or limited career opportunities [14]. Although 50% of cancer patients receive radiotherapy, the funding of radiation research appears to be rather low, and the opportunities may even be decreasing [15]. In addition, it has been shown that 50% of funded research projects are carried out within a rather small number of research organizations, raising the question of how a more nationwide funding of research projects can be achieved [16].

While working in radiation oncology can be highly motivating and fulfilling, this intersection of high-end technology and dedication to cancer patients also requires a highly trained and skilled workforce to maintain a high level of quality [17]. While a number of surveys have reported high levels of satisfaction and a comfortable working environment in radiation oncology, other sources report an exponentially growing shortage of well-trained specialists, as driven by generational change and the retirement trend of the baby boomer generation [18, 19]. Leading radiation oncologists from teaching hospitals report problems with finding medical staff almost 50% of the time [20]. Recent data suggest a potential shortage of about 18% for radiation oncologists in Europe overall [21, 22]. This means that the challenge of the future lies in inspiring and retaining young talents for the various professional specialties. Thus, factors that may discourage specialists from entering the field need to be identified and addressed by the radiation oncology community in order to maintain its independence from other departments and to remain a highly competitive medical specialty in its own right, without being incorporated into other specialties. One major challenge to acquiring sufficient numbers of young professionals is that each subspecialty has different requirements that have to be fulfilled to achieve job satisfaction. The aim of this study is therefore to examine the current situation of young professionals in all three subspecialties within the field of radiation oncology and to identify factors that may need to be addressed in order to provide a prosperous working and development environment for potential future professionals.

## Materials and methods

Within the framework of the young DEGRO (*Deutsche Gesellschaft für Radioonkologie*, German Society for Radiation Oncology) trial group and in close cooperation with the young DeGBS (*Deutsche Gesellschaft für Biologische Strahlenforschung*, German Society for Biological Radiation Research) and the young Medical Physicists (MP; *Deutsche Gesellschaft für Medizinphysik* [DGMP], German Society for Medical Physics), a web-based questionnaire with one general and three occupation-specific questionnaires for physicians, biologists, and medical physicists working in radiation oncology and radiation research was developed. The aim of the survey was to identify the needs and wishes but also the level of satisfaction of the young workforce working in radiation oncology and radiation research.

### Survey

An anonymous questionnaire was created using Survey Online® (Enuvo Inc., Switzerland). The survey consisted of a general part (13 questions) and 20–25 specialty-specific questions (physicians: 22 questions; biologists: 25 questions; medical physicists: 20 questions). 51 questions were mandatory (general: 9 questions; physicians: 14 questions; biologists: 17 questions; medical physicists: 11 questions), 29 questions were voluntary of which 17 were open-text questions (general: 4/3 voluntary/open-text questions; physicians: 8/4; biologists: 8/6; medical physicists: 9/4). The survey (for the full survey, see Supplementary Material 1) was designed with questions that allowed for single-choice or multiple-choice answers; some answers had an additional open-text field, and for the assessment of, e.g., workload or translational research opportunities, answers were ranked on a scale of 1 (very low) to 100 (very high) according to the participant’s subjective opinion. Free-text replies of participants were used to identify common problems and possible solutions. These insights from free-text replies were used to identify the most prominent questions, which are addressed in the Discussion. Prior to publishing the survey, a 3-day test phase was initiated, during which representatives of the subspecialties filled out the survey in order to identify potential shortfalls and problems. The survey was available for 6 weeks, from 22 June 2023 to 2 August 2023.

### Distribution

Initial distribution of the questionnaires took place during the 29th Annual Meeting of the DEGRO in Kassel, Germany, on 22 June 2023. This was followed by distribution via the young DEGRO, young DeGBS, and young MP mailing lists, telephone calls, social media posts, and emails to various representatives from 42 (university) hospitals and 6 research institutes in Germany.

### Cohort and exclusion criteria

As the survey was distributed at a conference, via social media, and via emails to various representatives, it is not known how many young academics were reached. In addition, 13% (Supplementary Table 1) of participants were aged 40 years or older, who are often not considered to be part of the young workforce. However, even those aged 40 years and older are expected to work for at least another 25 years. We thus chose to include this age group in the survey as, for example, having children or changing fields could result in a higher age while still holding a young professional position. Indeed, 27 of 34 participants from the 40 years and older age group also said that they were taking care of children or family members. Thus, all participants were included in the analyses provided they had completely filled out the survey.

### Statistics

Statistical analysis was carried out using GraphPad Prism© (GraphPad Software, LLC, version 9.5.1, Boston, MA, USA). Descriptive statistics were used to visualize the answers given by the participants. For graphs showing the median distribution of the participants’ opinions, data are expressed as median + interquartile range (IQR; 75% percentile–25% percentile).

## Results

### Participants

**Fig. 1:**
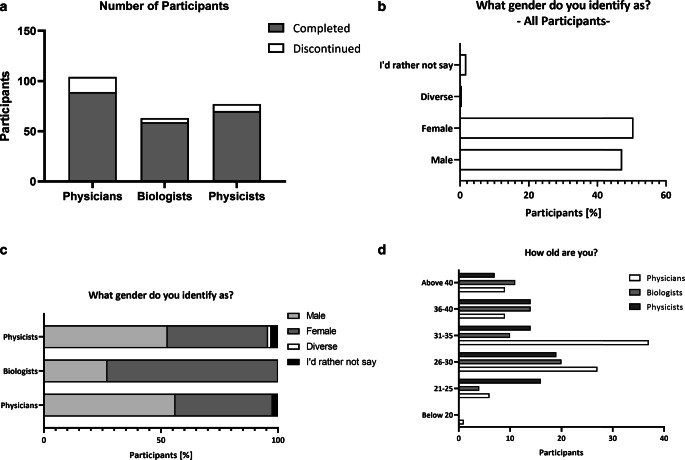
Overview of participants’ characteristics. Number of discontinued and completed surveys (**a**) and the percentages of identified genders summarized for all participants (**b**) and in terms of the specific occupation (**c**). Age distribution is shown in absolute numbers and divided into subspecialties (**d**)

Figure 1 gives an overview of the participants. While physicians in general are the largest group in the field of radiation oncology, there was also high participation of other subspecialties. Despite being the smallest group, biologists showed relatively high participation (*n* = 59) compared to the larger groups of physicians (*n* = 89) and (medical) physicists (*n* = 70; Fig. 1; Supplementary Table 1). In terms of age distribution, the cohort of biologists and physicists displayed a relatively even age distribution, while the participating physicians exhibited a peak between the ages of 26 and 35. While the gender distribution was balanced (51% female, 47% male), an examination of the subgroups showed a higher participation of females in biology (43 out of 59), whereas participants in the physicist (30 out of 70) and physician cohorts (37 out of 89) were predominantly male. An overview of participants’ characteristics can be found in Supplementary Table 1, Supplementary Material 2.

### Occupation-specific characteristics of participants

**Fig. 2:**
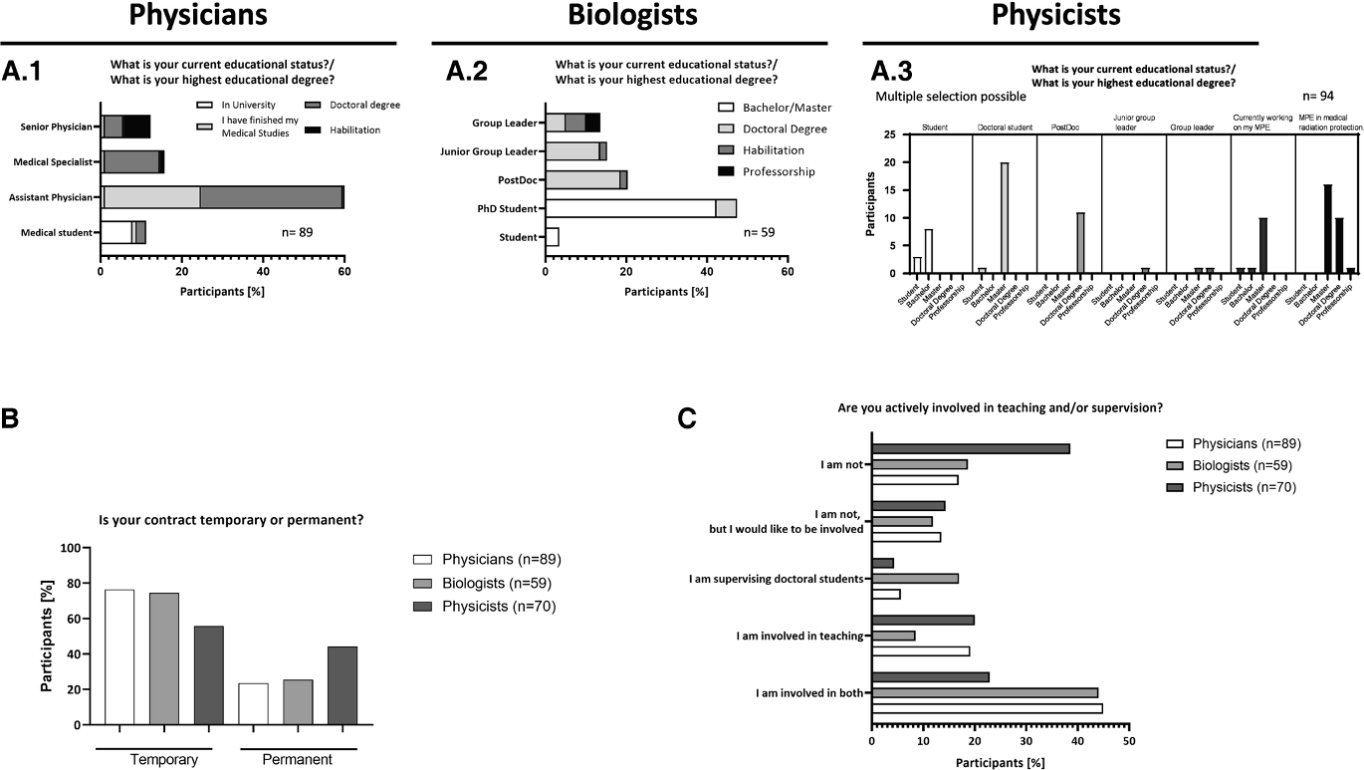
Occupation-specific characteristics of participants. Graphs (**A**) show the current educational status of participants, combining current educational status as well as degree. (**B**) Indicates percentages of temporary and permanent contracts within the subspecialties, while (**C**) shows the percentage of participants actively involved in teaching

While the majority of participating physicians were assistant physicians with a doctoral degree, the majority of participating biologists were PhD students with a bachelor’s or master’s degree (Fig. 2A.1 and A.2). Among the participating physicists, a large number of participants were certified medical physics experts (*Medizinphysikexperte*, MPE) with a master’s or doctors’ degree or postdocs with a doctor’s degree and master’s students working on their master’s degree (Fig. 2A.3). Regarding the type of employment contract, the vast majority of biologists (74.6%) and physicians (76.4%) held temporary contracts, while the physicists were more likely to hold a permanent contract (44.3%; Fig. 2B). When asked about their involvement in teaching and supervision, the physician and biologist cohorts were more involved in teaching and supervision than the physicist group, where 38.6% replied that they were not involved in teaching at all (Fig. 2C). Biologists are the group most involved in supervising doctoral students (17%; Fig. 2C), while physicians and physicists are more involved in teaching (19.1% and 20%, respectively). All three groups are equally willing to increase their involvement.

### Level of satisfaction and future perspective in the younger workforce

**Fig. 3:**
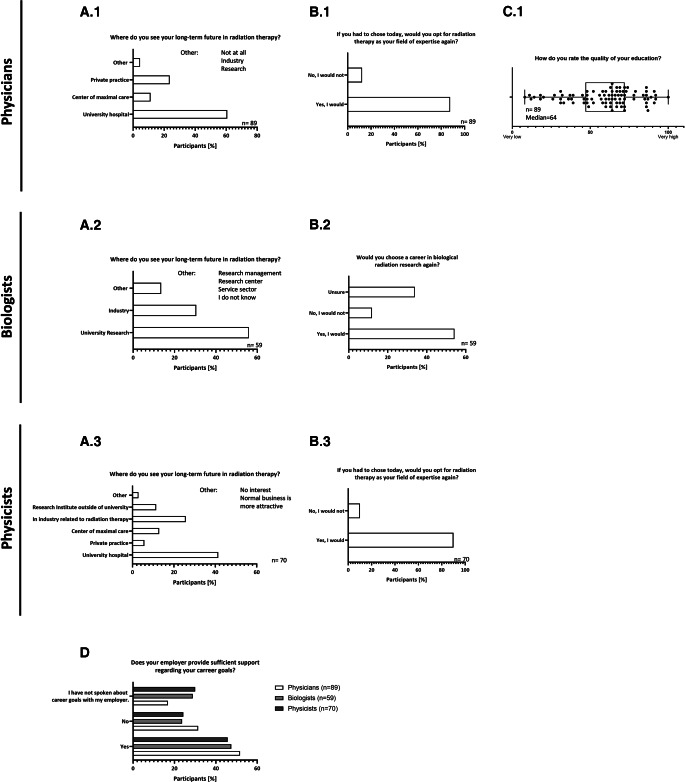
Level of satisfaction and future perspectives among the younger workforce. Shown are the percentages of the responses to questions about the participants’ long-term future in radiotherapy, with common free-text responses added to the top right of (**A**). Percentages of whether or not participants would choose a career in radiotherapy/research again are shown in (**B**). As the quality of education is a subject of great interest in the medical field, this question was only asked in the subgroup of physicians. Participants were asked to rate their perceived quality of education on a scale of 1 (very low) to 100 (very high), and the results are depicted as median, with individual responses represented as dots (**C**). We also asked whether participants received sufficient support from their employer (**D**)

In our survey, university hospitals were found to be the most attractive employer (physicians 60.7%, biologists 56%, physicists 41.4%; Fig. 3A.1 to A.3). For physicians, private practices seem to be more attractive (23.6%) than employment at centers of maximal care (11.2%; Fig. 3A.1). Only around 5% of the physician participants envision a future outside of radiation oncology. While physicists, due to the nature of their profession, have the most opportunities per se, the numbers of physicists inclined to work in industry (25.7%; Fig. 3A.3) is equal to the number of biologists willing to work in industry (30.5%; Fig. 3A.2).

While the vast majority of physicians (87.6%) and physicists (90%) confirmed their decision to enter the field of radiation oncology (Fig. 3B.1 and B.3), the biologist cohort has a large number of participants who are unsure of whether they would choose the same work field again (33.9%; Fig. 3B.2). The majority of physician participants rated the quality of their educational training as above average, with a median rating of 65 (IQR: 72–47) on a scale ranging from 1 (very low) to 100 (very high), as seen in Fig. 3C.1. In terms of subjective support in pursuing career goals, all cohorts report similar rates of lack of support (23.7 to 31.5%; Fig. 3D), with physicians taking the lead with a proportion of approximately 32% (Fig. 3D), while being the smallest group of participants not having spoken about the career goals with their employer (16.9%). Both physicists and biologists report a lack of communication about career goals in one third of cases. Approximately 50% of participants (45.7% to 51.7%) from all specialties report sufficient subjective support in achieving career goals.

### Concerns of all subspecialties about the future among the young workforce

**Fig. 4:**
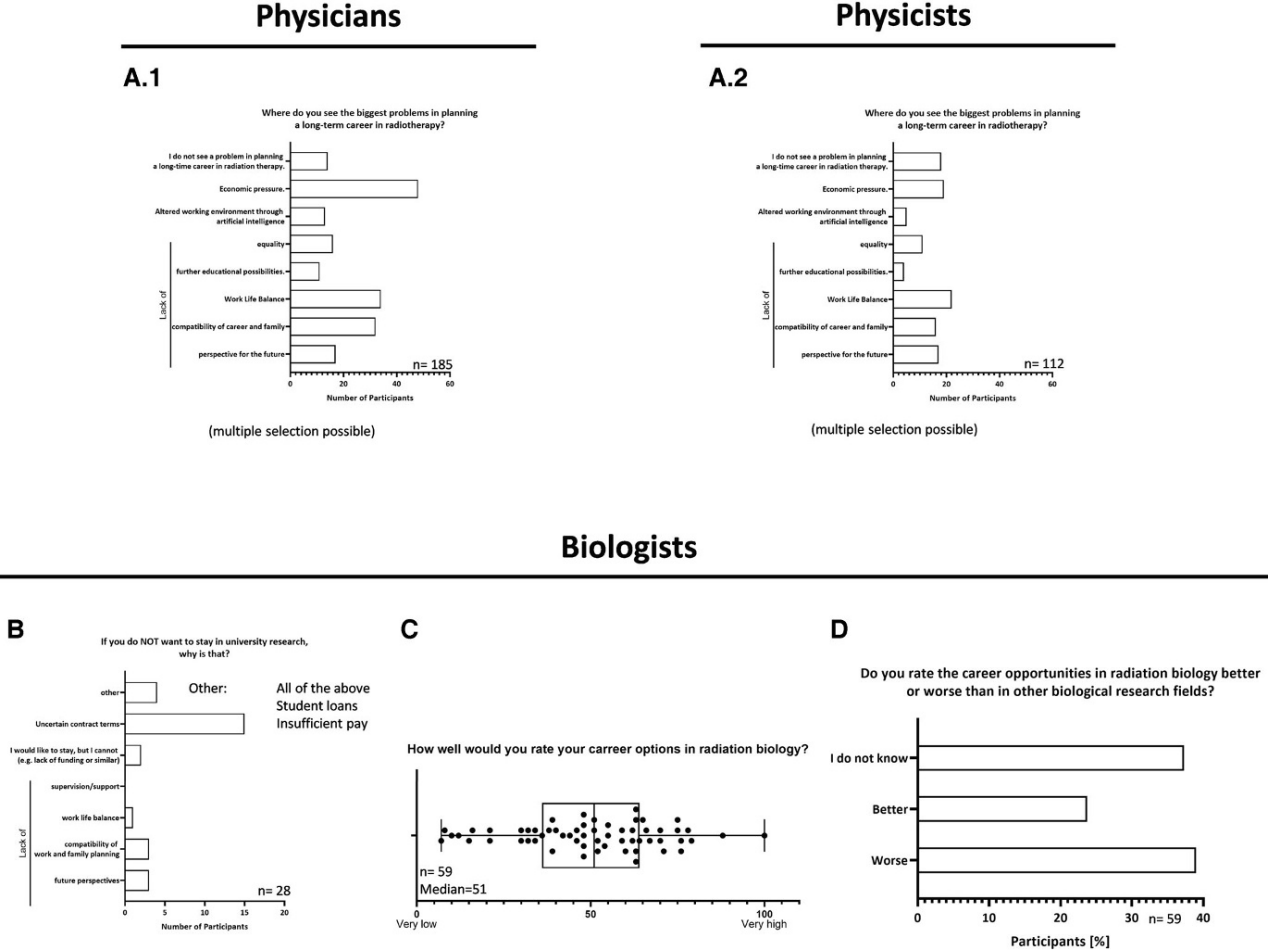
Concerns and considerations of all subspecialties about the future among the young workforce. Figure (**A**) Absolute numbers in answers given with regards to problems affecting radiotherapy as a long-term career goal (multiple answers were possible). (**B**) Absolute numbers of reasons why biologists exclude university research as a future work field (only those answering that they would not like to stay in academic research were asked to reply to this question). Participants were asked to rate their career options in radiation biology on a scale of 1 (very low) to 100 (very high); results are depicted as the median, with individual answers represented as *dots* (**C**), followed by the percentages of answers given to the question regarding whether career opportunities in radiation biology are perceived as better or worse than in other fields among the participants (**D**)

Three major concerns seem to be the leading causes limiting long-term career planning of physicians in radiotherapy: 1. economic pressure (*n* = 48) as the biggest problem in pursuing a long-term career among all medical participants; 2. work–life balance (*n* = 34); and 3. compatibility of career and family (*n* = 32; Fig. 4A.1). For biologists, uncertain contract terms (*n* = 15) seem to be the major concern when pursuing a career in radiation research (Fig. 4B). Further issues are very mediocre career options in the field (median: 51; IQR: 64–36; Fig. 4C) and a significant cohort even reports perceived worse career opportunities in radiation research than in other areas of biology (39%; Fig. 4D). For the participating physicists, the impeding factors seem to be a lot more varied, while the lack of work–life balance (*n* = 22) and economic pressure (*n* = 19) are also leading causes for not choosing a long-term career in radiation oncology (Fig. 4A.2).

### Subjective workload and weekly working hours

**Fig. 5:**
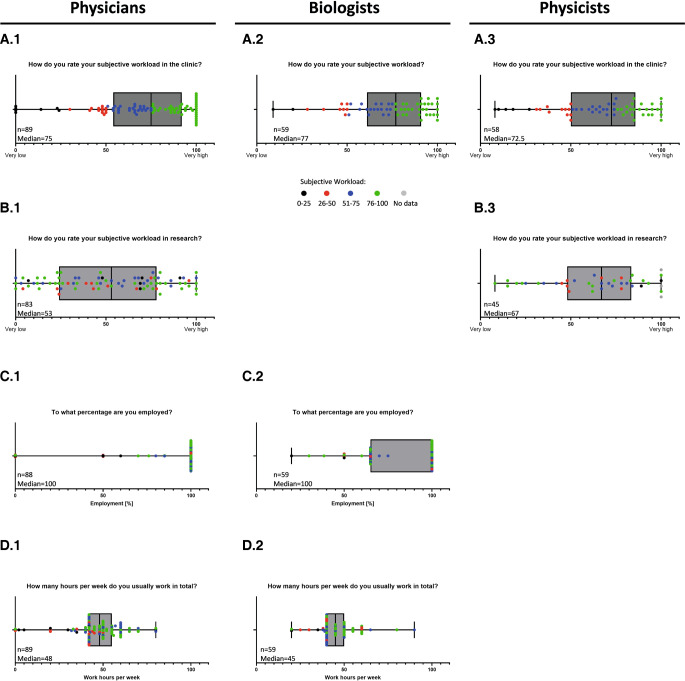
Subjective workload and weekly working hours in the young workforce. Participants were asked to rank subjective workload in the clinic or in general (**A**) and in research (**B**) on a scale of 1 (very low) to 100 (very high); results are depicted as the median, with individual answers represented by *dots*. As biologists usually do not have extensive clinical responsibilities, their subjective workload was not specifically separated into clinical work and research. It was requested that the percentage of employment (**C**) and working hours/week (**D**) be given in free-text form and these answers are depicted as the median with individual answers represented by *dots*. For a better comparison, part **A** was used to define four groups of subjective workloads (*0–25* black; *26–50* red; *51–75* blue; and *76–100* green). The assigned colors were kept consistent for the entire figure and for graphs **B**, **C**, and **D**

For physicians and physicists working in daily patient care, the weekly workload appears to be very high (physicists: perceived median of 72.5 on a scale of 1 to 100; IQR: 85.5–50; Fig. 5A.3), with physicians having the highest subjective workload (median: 75; IQR: 92–5; Fig. 5A.1). The perceived workload for research is more evenly distributed (median 53 for physicians; IQR: 78–24 and 67 for physicists; IQR: 83.5–48; Fig. 5B.1 and B.3). Regarding the differences between full- and part-time employment, the survey results reveal that physicians are mostly employed full-time, while biologists have a higher percentage of employees working part-time (Fig. 5C.1 and C.2). In terms of the number of hours worked per week, physicians lead the field with a median of 48 h (IQR: 55–42; Fig. 5D.1), while biologists reported a median of 45 h (IQR: 50–40; Fig. 5D.2). There are no data for physicists, as physicists who participated in the original design of the survey had decided not to include these questions in the questionnaire.

**Fig. 6:**
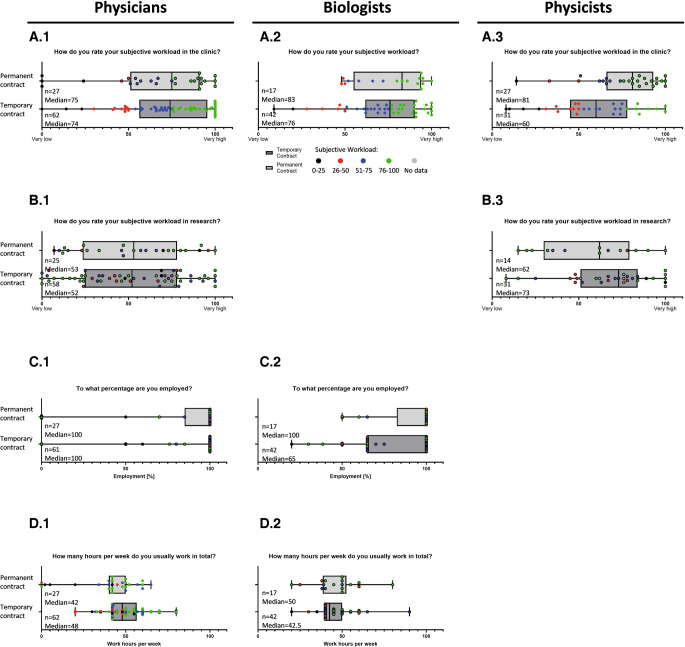
Subjective workload and weekly working hours in the young workforce depending on permanent and temporary contracts. Participants were asked to rank their subjective workload in the clinic or in general (**A**) and in research (**B**) on a scale of 1 (very low) to 100 (very high); results are depicted as the median, with individual answers represented by *dots*, and results of those holding permanent contracts depicted in *light grey*, while temporary contracts are shown in *dark grey*. As biologists usually do not have extensive clinical responsibilities, their subjective workload was not specifically separated into clinical work and research. It was requested that the percentage of employment (**C**) and working hours/week (**D**) be given in free-text form and these answers are depicted as the median, with individual answers represented by *dots*. For a better comparison, part **A** was used to define four groups of subjective workloads (*0–25* black; *26–50* red; *51–75* blue; and *76–100* green). The assigned colors were kept consistent for the entire figure and for graphs **B**, **C**, and **D**

The distinction by contract type (temporary vs. permanent) shows no difference in subjective workload among physicians (median: 74 vs. 75; IQR: 95.5–56.3 vs. 91–51; Fig. 6A.1 on a scale of 1 to 100), while there seems to be a trend towards a greater subjective workload for staff on a permanent contract among biologists (median: 76 vs. 83; IQR: 90.3–61.8 vs. 94–55; Fig. 6A.2) and physicists (median: 60 vs. 80; IQR: 78–45 vs. 93–66; Fig. 6A.3). While the subjective workload in research does not differ for physicians (median: 52 vs. 53; IQR: 78–24.8 vs. 78–23.5; Fig. 6B.1), the subjective workload in research appears to be higher for employees with temporary contracts among physics (median: 73 vs. 62; IQR: 84–51 vs. 79.3–29.8; Fig. 6B.3). While full-time employment seems to be the rule for physicians, with very few exceptions, the biologist cohort shows a wider distribution of working time models and more employees working part-time (Fig. 6C.2), potentially due to the high numbers of participating doctoral students (Fig. 2). This can also be seen from the fact that the median employment rate among temporarily employed biologists resembles 65% (IQR: 100–65), while the median employment percentage among those working on permanent contracts is 100% (IQR: 100–82.5; Fig. 6C.2). For the cohort of physicians, the median number of working hours per week seems to become smaller with a permanent contract (median 42 permanent contract vs. median 48 with a temporary contract; IQR: 50–40 vs. 56.5–42; Fig. 6D.1), while the opposite seems to be true for biologists (median 50 vs. 42.5; IQR: 52.5–38.5 vs. 50–40; Fig. 6D.2).

### Problems of subspecialties in day-to-day research work

**Fig. 7:**
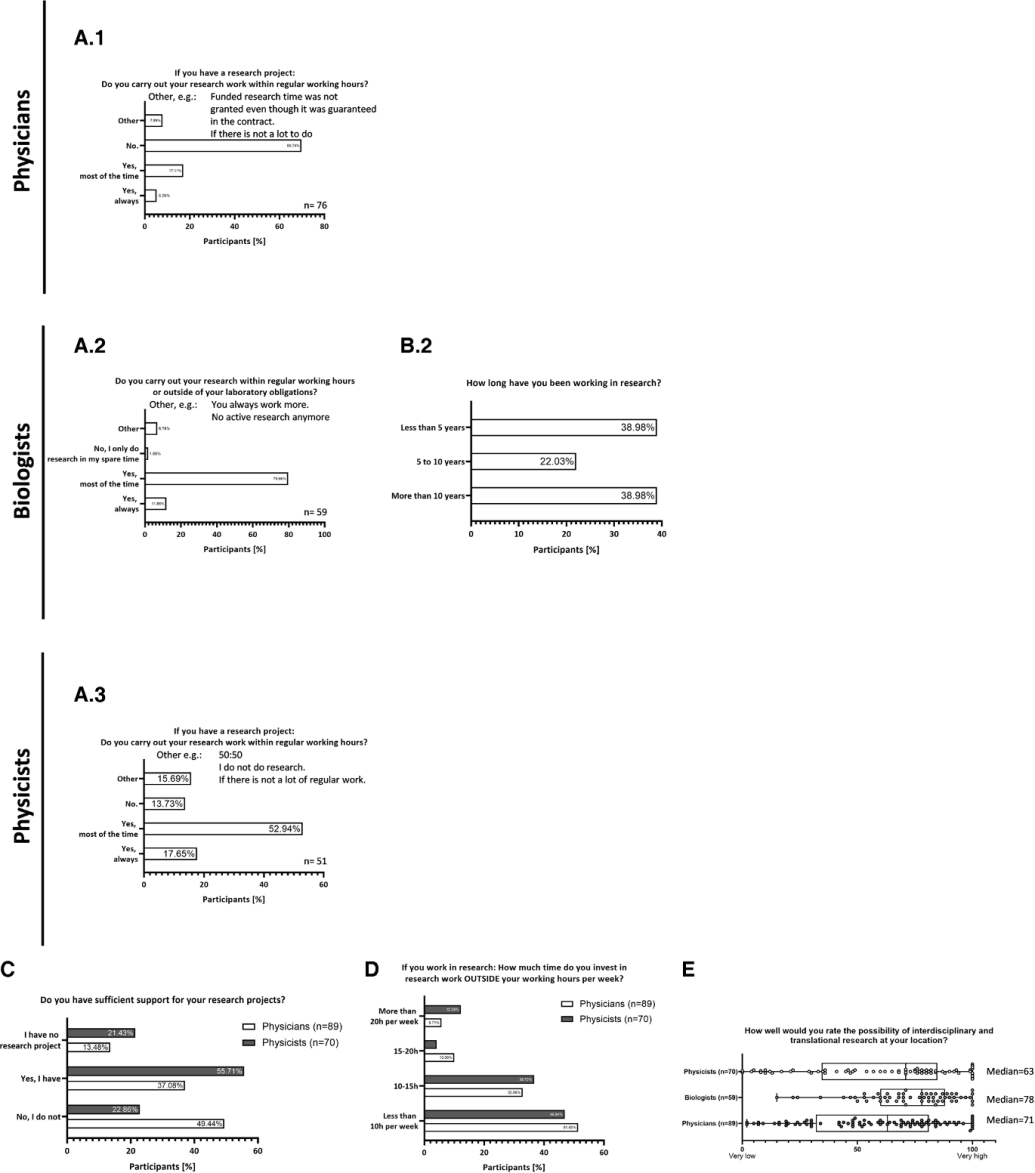
Problems in day-to-day research work. Part (**A**) depicts the percentage of research that is carried out during regular working hours, while biologists were asked how long they had been working in research; answers are given in percent (**B**). Part (**C**) shows the percentage of perceived support in research projects, while part (**D**) visualizes the number of hours spent on research outside of the regular working hours. For part (**E**), participants were asked to rate the possibility of interdisciplinary research at their location on a scale of 1 (very low) to 100 (very high); results are depicted as median, with individual answers represented by *dots*; answers are given in percent

Concerning research time distribution, approximately 70% of physicians do not conduct research during their regular working hours, with 17% reporting that they conduct research during regular working hours most of the time. Only 5% reported doing research strictly during their working hours (Fig. 7A.1). This means that 50% of the participants invest less than 10 h per week in research while 30% manage to invest 10–15 h and only 10% are able to invest 15–20 h in research. Only 5% of physicians invest more than 20 h per week outside of their regular working hours (Fig. 7D). While only a small percentage (13.5%) of physicians report not having a research project, only 37% report receiving sufficient support for their research projects, while 50% report a lack of support for their research projects (Fig. 7C).

On the other hand, physicists seem to be less involved in research projects (“I have no research project”: 21.4%; Fig. 7A.3), while more often receiving sufficient support for their research projects (55.7%; Fig. 7C). This is reflected by the fact that 53% of physicists carry out their research mostly during their regular working hours and 18% report that they only do research during their working hours (Fig. 7D). The cohort of biologists has 80% of participants doing research mostly during regular working hours, while 12% of the biologists carry out research strictly during regular hours only (Fig. 7A.2). Furthermore, 55.9% of biologists also reported that their tasks are not limited to the contractually agreed job description (data not shown).

The perceived ability to carry out interdisciplinary and translational research at their institutions rated on a scale of 1 to 100 is lower for physicians (median: 63; IQR: 81–32; Fig. 7E) than for biologists (median: 78; IQR: 88–60; Fig. 7E) and physicists (median: 71; IQR: 84.8–34.5; Fig. 7E). In particular, among physicians and physicists, there appears to be a very broad distribution of perceived opportunities for translational research at their respective locations, thus lowering the overall median, whereas for biologists, there appears to be a greater presence of translational research.

### Participation and satisfaction with respective to professional societies

**Fig. 8:**
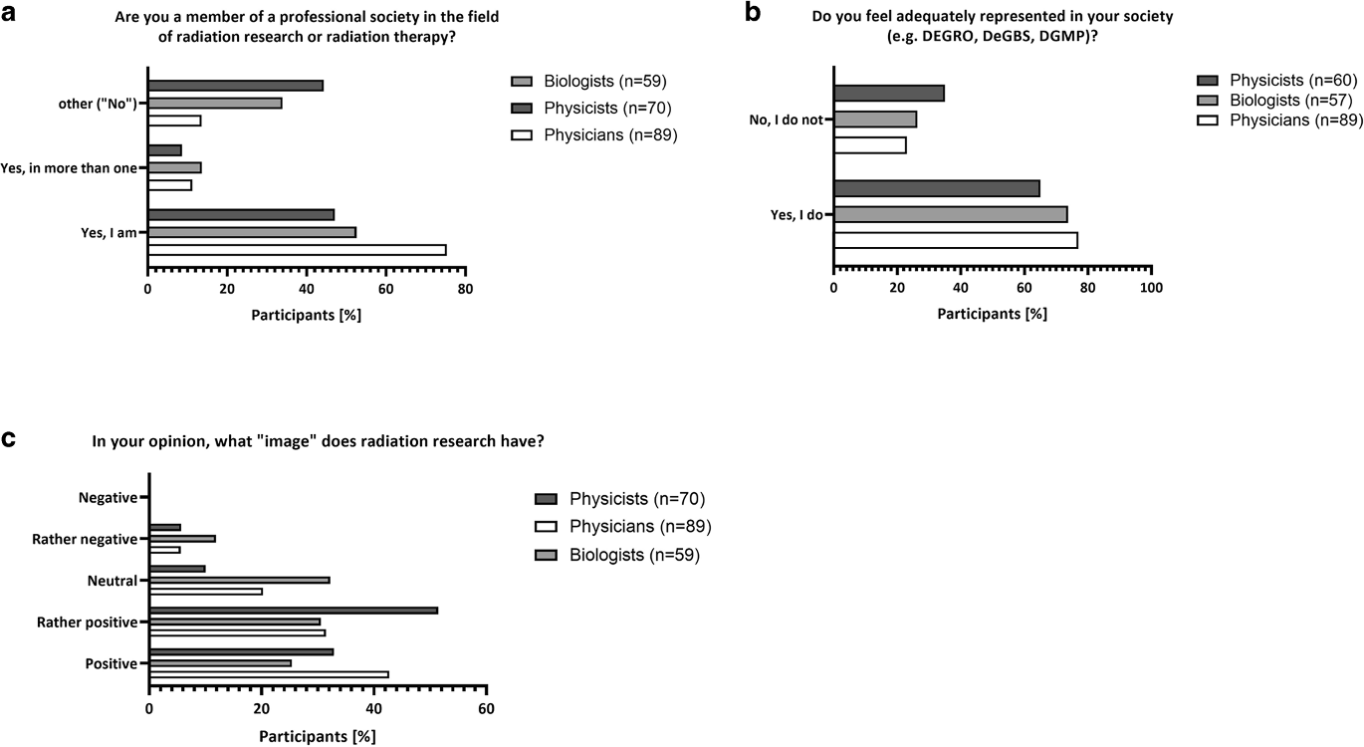
Participation and satisfaction with the respective professional societies*. *Participants were asked to indicate whether they are a member of one or more professional society. Results are given in percent (**a**). In order to better understand why, participants were further asked to judge how well they feel represented by their respective society (**b**), which is depicted in percent. Finally, participants were asked about the “image” of radiation research according to their individual perception. Results are indicated in percent (**c**)

Physicians show the highest rate of membership in a professional society (86.5%; Fig. 8a). Biologists have the highest number of participants in more than one society (13.6%; Fig. 8a). Physicists show the lowest number of participants in a professional society overall (44.3%; Fig. 8a) and feel inadequately represented by a society in 35% of cases (Fig. 8b). While the majority of physicians and biologists feel adequately represented (77% and 73.7%, respectively), around one quarter of participants still do not feel adequately represented by a society overall (Fig. 8b).

Regarding the image of radiation research, physicians in particular have a positive perception of radiation research (positive: 42.7% and rather positive: 31.5%; Fig. 8c), while physicists (positive: 32.9% and rather positive: 51.4%; Fig. 8c) and biologists (positive: 25.4% and rather positive: 30.5%, Fig. 8c) are more reserved. Biologists are most likely to see the image of radiation research as neutral (32.2%) or even rather negative (11.9%). None of the participating professions indicated that radiation research is perceived negatively.

## Discussion

Identifying the issues faced by the young workforce is an essential step towards improving the satisfaction of all subspecialties and improving the willingness to not only get involved but also to continue an active career in the field of radiation science. Despite physicians comprising the largest of the subspecialties according to the overall number of employees in Germany, it was especially biologists and physicists with a very high participation in the survey [2]. The overall high involvement during a rather short period of 6 weeks in comparison to similar studies [23, 24] indicates that there is potential, ambition, and interest among the younger employees to identify problems and improve the field of radiation oncology and radiation research over the next decades.

Our study revealed that each of the subspecialties has distinct problems (e.g., the contractual situation for biologists), while other factors are present cross-disciplinarily and thus need to be addressed by the societies as a whole.

### Subspecialties specific problems

#### Biologists

Radiation biology, despite not being present in all departments, makes a tremendous contribution to the field of radiation oncology and radiation protection by providing knowledge of dose fractionation, tumor hypoxia, tissue weighting factors, and radiation quality [25, 26] as well as regarding the optimized use of combination therapies. Radiation biologists have helped to define parameters needed for risk and exposure assessment, thus establishing dose limits and offering mechanistic explanations for outcomes observed in radiation epidemiology that provide the pivotal translation of laboratory data into human applications. Insights gained by radiation biologists are used in clinical work for radiation protection, diagnosis, risk assessment, and treatment. While our survey reports that most participants see only mediocre career options and about 40% even worse career opportunities than in other biological fields (Fig. 4), one has to point out that the insights and knowledge gathered can be used not only in the public health domain but also for military purposes, space exploration, environmental stewardship, and national security, and also provide biological and biophysical information on radiation response to federal agencies setting exposure limits for workers and the general public. This can of course be used for creating job opportunities, and the gathered knowledge can be used as a transition into other scientific fields or jobs outside of the field of radiation oncology. In these cases, career seminars might be helpful in order to point out alternative career options for biologists working in radiation research. In this way, well-trained high-potential staff will not get lost to other disciplines or subjects but will find ways to remain in radiation research outside of the medical field. An uncontroversial fact is that securing research funding, which is often challenging to obtain, places a significant burden on employees, as it is an essential element for maintaining a position and providing the potential for career advancement, especially in biological research [14]. The probability of funding, on the other hand, is strongly determined by strong mentorship, establishment of a collaborative network of scientific and clinical expertise within the host department, and scientific input from related departments both within the host department and from external institutions. Besides typical support with data analysis, biostatics, literature, and article writing, intensified help in preclinical animal studies with exceptionally hard and complex legal and ethical regulations should be considered [9]. At the moment, the majority of postdocs are forced to cover their position using grant money, which basically makes them cheap, smart, and highly educated labor [27]. Short-sighted hyper-competitive environments potentially even slow down scientific innovation and productivity and favor fabrication and publication of immature data, thereby wasting valuable resources. High expectations and success—despite the low probability of success in the whole research environment, ranging from grant applications to hiring decisions and career progression—strongly depend upon publications, which encourages scientific dishonesty [28]. Improvement can be achieved by preparing trainees for different potential career paths, teaching training skills that can be used outside of the academic world, creating communication platforms, and giving postdocs more visibility and thus possibilities to be more proactive about their career. Another approach could be to optimize the postdoc salary with cost-of-living adjustments [29]. Improvements need to be made towards individual grant application by postdocs and improving grant evaluation for younger researchers with lesser-known names and reputations, thus enabling high-potential scientists to focus on research instead of writing grants [28]. The harsh working conditions are very well reflected by the low percentage of participants in our survey who would choose radiation biology again. In combination with the mediocre career options and even worse-perceived employment options overall compared to other biological research fields (Fig. 4), attracting high-potential biologists to the field is becoming more challenging. However, as a high number of individuals in our survey are unsure about whether or not to remain in radiation research, it is evident that adequate measures can aid in keeping these individuals rooted in radiation research.

#### (Medical) physicists

Physicists represent the second largest cohort of employees involved in the field of radiation oncology. A regulatorily required number of (medical) physicists is of utmost importance for the safety of patients and the quality of radiation treatment. Important tasks carried out by physicists range from administrative and clinical services to informatics, equipment maintenance, and performance evaluations, as well as research, teaching, and training. The past has shown that implementation of new techniques like intensity-modulated radiotherapy (IMRT) leads to an increased demand for physicists [19, 30, 31]. Despite being of such great importance, very few new academic positions are being created in research and education in health care physics and medical physics overall [32]. Nevertheless, the profession will likely continue to further increase in importance, mostly due to the vast amount of new technology that will need to be implemented into current treatment protocols. In the present survey, physicists show the highest willingness to leave academia and transition into industry, while private practices are the employers least favored by physicists, which could be biased as participants of the survey are mostly employed at university hospitals. However, the fear of less challenging and versatile cases as well as repetitive daily tasks in peripheral institutions could also be a reason for this. As well-trained physicists can easily transition into other work fields, it is an essential task to better include these specialists in the society and keep these individuals in the field of radiation oncology in order to remain competitive in the years to come, as inadequate staffing will inevitably lead to loss of quality and safety in the long run. One possible way to achieve long-term commitment could be an increase in the permanent contracts offered by institutions. Physicists represent the subspecialty with the highest percentage of permanent contracts in the survey, which seems to reflect the particular need for long-term integration of well-trained personnel into workflows of the respective departments to ensure consistency and reliability, but also the legal requirements for the presence of MPEs. It remains unclear why a large percentage of (medical) physicists are not members of a society (Fig. 8). It may be possible that bachelor’s/master’s students, due to their early career stage, and non-scientific MPs may not see any representation of interests in a scientific society. As there is a lack of a professional association for clinically working medical physics experts in Germany, the offer of (interdisciplinary) education and interaction for this group should eventually be further expanded within the existing scientific associations. At the same time, this may increase interest and active participation in the societies and strengthen interdisciplinary and translational cooperation between research and clinical settings. In general, the cohort of physicists seems to be rather satisfied with their career choice, reporting the highest percentage of participants who would choose the same specialty again and high satisfaction rates regarding the support offered by the employer (Fig. 3). Only the subjective workload in science tends towards being higher compared to the physician cohort.

#### Physicians

Physicians are the largest cohort and also represent the subspecialty with the highest visibility due to the direct patient contact. In our survey, the most prominent fear among physicians is high economic pressure followed by a lack of work–life balance and compatibility of career and family (Fig. 4). Regarding the general economic situation, lower financial reimbursement combined with higher costs for personnel, energy, and maintenance cause significant economic stress in the different departments. Profit targets set by the hospital administrations are a balancing act between the economic situation and a reasonable treatment for patients. In Germany, a large cohort of patients undergo treatment within private practices, which due to newer rules, regulations, and alterations in billing could possibly put those practices under significant economic pressure in the future, especially as insurance companies try to cut costs overall [4]. Another significant point defining employee satisfaction is the subjective or perceived workload. The present survey shows that especially physicians have the highest subjective workload among all subspecialties (Figs. 5 and 6). Recent studies report that a continuously high workload can lead to depression and loss of workforce in the long term, resulting in a negative public perception of the field [33–35]. As especially research work outside of regular working hours can contribute to the workload, protected research time needs to be implemented using specialized programs or research grants.

### Common problems in the younger workforce

#### Work–life balance

Another topic that needs to be addressed for all subspecialties in the future is the creation of a job environment with an adequate work–life balance, as this is one of the major concerns of the physician and physicist cohorts in our survey. Compatibility of career and family, especially with a higher number of female professionals (Fig. 1), paired with significant changes in classical family roles, is also increasing in importance. The possibility of working part-time is an essential factor, facilitating better adaptation to private circumstances such as nursing a family member or taking time out for childcare. Another possible solution is implementing the concept of a 4-day working week that is currently discussed [36, 37] and even being tested in some clinics in Germany. While the biologist cohort shows a fair number of employees working part-time, this is rather unusual for the physician group, which shows the highest rate of full-time employment (Figs. 5 and 6). However, as doctoral candidates in biology or medical physics are employed part-time while usually working full-time, an in-depth analysis of these factors will need to be carried out in the future. Thus, this is in most cases only a pro-forma part-time contract, while PhD students actually work full-time. True part-time contracts might, however, increase the attractiveness of the field through improved work–life or work–family balance. The high percentage of full-time-employed participants in the physician group could possibly be explained by the need of finishing a residency program as the final step of their education. In the future, more liberal part-time models should be offered and will be inevitable as more and more female employees strive into the field. Working part-time in general needs to become more accepted by supervisors independent of gender to enable contemporary gender-equal divisions of labor and non-classical family roles.

#### Working environment

The most favored form of employment in this survey is academia among all subspecialties (Fig. 4). One possible explanation could be that academia combines clinical, scientific, and teaching work, in contrast to non-academic clinics or private practice, and offers the chance to work in a cutting-edge and up-to-date environment that is usually less economically driven. University hospitals offer, besides a basic education, the possibility to acquire further scientific education, helping to implement new results into daily work as paradigm changes can occur very fast in modern medicine [38, 39]. However, the structure of universities overall will not make it possible for all participants to pursue a career in academia. Further considerations discouraging long-term academic careers include academic pressure, declining support for research, and increased bureaucracy [17, 40, 41], as also visible in the present study (Fig. 4).

### Potential points of improvement

A survey by Krug et al. about the young radiation oncology workforce showed that only 4% of participants reported a complete separation between clinical work and research and/or teaching activities. Among these, physicians reported the lowest number (9%) of protected time to carry out research or teaching in their regular working hours on a regular basis [2], suggesting that most of the time, teaching and research are carried out outside of regular working hours. This can potentially become problematic, as the quality of research and teaching but also of clinical work might suffer when tasks are mainly carried out outside of regular hours and during overtime [42].

#### Research

One possible point for improvement is the creation of easier options for entry into scientific work. While the volume and intensity of research will differ significantly between facilities, barriers impeding participation in research have to be clearly addressed. Obstacles that are frequently mentioned range from lack of time, limited access to statistical analysis through insufficient mentoring, and variable support from superiors, to great variability in research productivity at the department level, partially due to metafactors such as the size and composition of the department, advanced technology capability, limited access to funding, and the numbers of patients and degree of engagement with specialist medical research institutions [43]. The present survey confirms that especially employees in the physician cohort lack protected research time (Fig. 7). Combined with the fact that overtime in work covering the clinical routine is becoming the rule rather than the exception, this makes it virtually impossible for clinicians to engage in high-quality and pioneering research [4, 44]. Furthermore, according to our survey, physicians and physicists are less frequently involved in the supervision of doctoral students compared to biologists. This can be attributed to a higher level of clinical involvement and responsibilities; likewise, it is possible that these groups are more involved in the supervision of bachelor’s or master’s theses. As scientific work is time consuming, an approach to include mainly clinically working personnel in research, thus giving them an overview of current trends and developments outside of their daily work, could be to involve them in the reviewing process of scientific work after respective training. Other countries require medical candidates to complete mandatory scientific work before their final examination. In the case of clinical medical physicists in training, completion of a scientific project or an additional study before becoming eligible for certification could be obligatory—an approach that has been successfully implemented in the model medical study course in Cologne, Germany [45–47]. Another recent improvement in this regard is the introduction of young scientist scholarships, enabling physicians to take protected research time as a clinician scientist [48], which could significantly improve the situation. This is especially important as fundamental research on combining radiation therapy with other treatment modalities like surgery and chemo-/immunotherapy will become more and more important in the future. Intensifying translational work cooperations will be an inevitable component of this.

#### Teaching

Likewise, lectures on physics and biology have to be implemented into standard residency programs to generate a basic understanding of the mode of operation of, e.g., immunotherapies, and to foster awareness of possible cooperations and interactions between subspecialties [49]. Furthermore, the satisfaction of trainees with their occupational training is of utmost importance for choosing a certain career path. Participants from the medical field that were asked about their educational quality in our study, however, report a broad range of satisfaction, with a median of 65/100 (Fig. 3). There is a significant number of participants that seem to be highly satisfied with their education versus 29% of participants reporting satisfaction rates of below 50. One option could be to hold written and oral examination on the topics of radiation biology and radiation physics early during residency, following the example of the radiation protection course. These results could also give more feedback to the teaching institutions from an educational standpoint [50]. Cooperations between biologists and physicists also need to be intensified, as new radiation-generating devices show increasing complexity, requiring formal training in basic principles of radiation dosimetry and measurement for research.

Our survey further reports that the involvement of physicians and biologists in teaching is higher than that of the subcohort of physicists (Fig. 2). While medical physics most likely will continue to grow in importance due to increasing numbers of cancer cases as well as further scientific improvements leading to advanced technologies that increase the demand for well-trained professionals [51], the high number of participants not involved in either teaching or scientific work is problematic and needs to be improved in the future.

#### Mentoring and networking

One quarter of participants indicate insufficient support for their career goals, potentially causing them to leave the field in the long term. Among the participants, the cohorts of biologists and physicists seem to be a lot less prone to communicating their potential career goals to their employers (Fig. 3), which could be interpreted as either being less career oriented or as being less informed regarding the possibilities in the field. Here, career courses could be an option to inform and train staff to better identify and communicate their goals. Another possibility might be a widespread implementation of mentoring programs that pair young professionals with those more experienced in the field who often have a more widespread network and can offer pivotal guidance in personal, clinical, and academic growth. Especially mentoring has been frequently mentioned during the current survey. The supervision by mentors could help to create a meaningful timeline for reaching certain milestones and complementary educational achievements such as a basic education in health care economics, as often required for higher positions [52], especially as mentors can give advice independently of the department’s own interests. Recently, the young scientist groups have reacted to this topic and offered a mentoring program for all three subspecialties involved in radiation oncology, highly supported by older members of the society in Germany.

### Future developments and generational change in radiation oncology

Another point that needs to be discussed is how the clinical demand will change as a response to growth in the cancer incidence (approximately 2.5% per year), the impact of new technologies with possible acceleration of treatment-related workflows due to artificial intelligence, but also possible slowing of processes due to more adaptive and individualized treatment regimens [3]. While future developments cannot be entirely foreseen, technical progress, which holds great potential for improvements in treatment overall, is not seen as a significant problem, neither by the physicist nor by the physician cohort (Fig. 4). In contrast, new technologies can even lead to an increase in treatment time. Models and further predictions show that a 5% increase in overall workload could potentially lead to a significantly higher number of staff needed from all involved specialties [3]. One example is the increase in demand for medical physicists due to the introduction of new technologies and an increased number of treatment machines [19, 53, 54]. Combined with a general shortage of personnel due to aging of the existing workforce, which can only be compensated to a very limited basis, this could lead to a real decline in treatment capacity, safety, and overall quality. Limitations could be reduced through the extension of work contracts or consulting and by increasing the number of open clinical training positions for MPEs [30, 53, 54].

While a lack of potential employers is present for a large variety of specialties, overall competition for motivated and well-trained employees due to suboptimal working conditions and payment level compared to some other European countries is inevitable. Radiation oncology will probably not be given more attention in undergraduate life science education, thus limiting its visibility to potential future employees at that early stage of their career path. Therefore, advertising the field by offering good and diversified training programs with a wide range of additional offers could serve as a letter of invitation, convincing potential candidates to take their decision in favor of radiation oncology in the end. In contrast to a lack of potential employers, the generational change benefits investor-managed hospital and practice chains primarily focusing on increasing margins rather than prioritizing improving patient treatment.

Radiation oncology is underrepresented in oncological teaching in medical schools, which leads to underutilization and a lot of prejudices towards the field, while studies show improved knowledge with dedicated courses, especially favoring clinical exposure over dedicated lectures [37, 55, 56]. The lack of knowledge potentially even impedes multidisciplinary oncological care. Furthermore, a recent study demonstrates that the introduction of radiation oncology into the preclinical part of medical education is feasible [57], an idea that is generally well perceived by students [58]. This may aid in attracting more doctoral candidates and residents while improving overall knowledge in radiation oncology. Possibilities to teach radiation oncology outside of the existing curriculum include special lectures, seminars, and bedside training as well as the meaningful integration of e‑learning [59]. Teaching in biology and medical physics is generally less frequent [24]. The decision to choose a certain specialty is driven by different intrinsic (personal interest) and extrinsic (perceived prestige or potential income) as well as structural considerations like the influence of lifestyle, working hours, perceived stress from on-call duties, amount of time on-duty, and the possibility to work part-time. Although no one single factor can be seen as decisive, a beneficial combination of exactly these points could give the field of radiation oncology the edge, especially for students still undecided at the end of their studies [60]. Awareness and basic knowledge in these fields, however, also needs to be established amongst general practitioners to provide patients with adequate advice and information [24].

### The role of societies

Another promising step towards improvement is the creation of networks among younger professionals within the framework of existing societies. The creation of internal groups linking young radiation oncology scholars has been successfully implemented in other European countries [9]. Structured collaboration between new and advanced professionals within the society can further address essential points such as the drafting of a good residential curriculum, including reliable long-term rotation plans covering all relevant aspects of clinical radiation oncology and naming individuals responsible for the supervision of residency training. Another possibility would be the creation of improved teaching including lecture series covering all the major relevant topics in oncology in a location-independent approach, ensuring that all residents can benefit from the knowledge of local specialists. Such a step was recently taken by the young DEGRO in Germany and has been met with a very positive response [42, 61].

Teaching, which is often not standardized throughout the different departments, has been further compromised by the COVID pandemic [42]. The young DEGRO group has implemented online courses covering different topics in physics, biology, and clinical radiation oncology. The courses so far have been very well received, inviting new aspiring scientists and clinicians to present a talk on their research focus. A similar program is found in the DGMP and young MP, while the young DeGBS focuses on online seminars covering topics such as how to set up a lab or introduction of committees. Points that have to be further addressed include more time for self-study; increasing support by the DEGRO, DGMP, and DeGBS; participation in meetings; improved educational quality; and an institutional training schedule that focuses more on the guidelines drawn up by the German medical council [61, 62].

Another viable option could be the creation of a central research and teaching team organized by the DEGRO and young DEGRO in collaboration with the DeGBS and young DeGBS as well as the DGMP and young MP to assist in the setup of studies and offer help with valid statistical evaluation, research training, and the advancement of science overall [63]. Another more decentralized possibility could be the creation of a core research team in each university clinic consisting of a clinical researcher, a biologist, and a physicist with clearly defined tasks and protected time for the generation and supervision of larger-scale studies as well as assisting in translational research approaches. More ambition in this field by DEGRO/young DEGRO could improve the number of members of all subspecialties in the society, as there will be an improved sense of personal benefit from joining the society ([17]; Fig. 8).

In general, giving more power to young societies not only creates opportunities for getting involved but also allows for more uniform training, also including special techniques such as new imaging modalities (for example, PET-CT, MRI, special sequences) as well as insights into less mainstream areas of radiation oncology such as brachytherapy.

### Limitations

As this is the first study combining a team of young DEGRO, young DeGBS, and young MP members, the present interrogation could not rely on a validated questionnaire but was composed of newly developed questions, thus leaving room for multiple interpretation possibilities that need to be further specified in the future. Nevertheless, this approach was chosen due to the nature of the survey at hand. We have included a wide number of occupations, workplaces, and research foci in this survey, as all of these professionals work in radiation oncology and radiation research and thus also have a wide range of specific occupational requirements. This approach thus allowed generation of a broader overview. Further studies will also have to include the specific needs of radiotherapy technologists, especially with regard to the declining numbers of this essential profession.

## Conclusion

The possibility of collaborating with different members of the radiation research community with diverse interests and expertise, the breadth of career opportunities, and the ability to adapt research to human needs are great strengths of the field [64]. All factors that may discourage applicants from entering the field have to be identified and successively addressed to maintain a competitive field and independence from other departments. As pointed out by Professor Zietman, former president of the American Society for Radiation Oncology (ASTRO), there is a danger that, if radiation oncologists become simple guardians of a single therapeutic modality, as time goes by and techniques live on and improve, the specialty as a whole may not. This, however, requires a proactive attitude on the part of all the participants in the field of radiation oncology [39]. Furthermore, it is the responsibility of researchers to engage a broader audience, illustrating the potential value they can provide to society.

## Supplementary Information

Supplementary Material 1 Complete online survey

Supplementary Material 2 Table 1. Characteristics of survey participants (*n* = 218). Absolute numbers are given in brackets. Numbers may not add up to 100% due to rounding error or missing values. Abbreviations: *d* diverse; *f* female; *m* male; *n/a* not applicable.

## Data Availability

The data presented in this survey are available from the corresponding author upon reasonable request in an anonymized manner.
