# Supplementary material for: German radiation oncology’s next generation: a web-based survey of young biologists, medical physicists, and physicians—from problems to solutions
Source: Strahlenther Onkol. 2024 Oct 22;200(12):1005–24. doi: 10.1007/s00066-024-02305-8 (PMC11588816; doi:10.1007/s00066-024-02305-8)
Supplement: Supplementary file 1 — Supplementary Material 1 Complete online survey [file 66_2024_2305_MOESM1_ESM.pdf]

Strahlentherapie und Onkologie

German Radiation Oncology's Next Generation: A Web-Based Survey of Young Biologists, Medical Physicists, and Physicians—From Problems to Solutions

Thomas Weissmann [1,2,3,†], Lisa Deloch [1,2,3,4,†,*], Maximilian Grohmann [3,5], Maike Trommer [3,6,7], Alexander Fabian [3,8], Felix Ehret [3,9], Sarah Stefanowicz [3,10], Alexander Rühle [3,11,12], Sebastian Lettmaier [1,2], Florian Putz [1,2], Maya Shariff [1,2], Simone Wegen [3,13], Johann Matschke [3,14, 15], Elena Sperk [16,†] and Annemarie Schröder [3,17,18,†]

† contributed equally

* Corresponding author: Dr. Lisa Deloch, Radiation Osteoimmunology at Translational Radiobiology, Department of Radiation Oncology, Uniklinikum Erlangen, Germany; lisa.deloch@uk-erlangen.de;

Supplementary Material 1

Complete Online Survey

Analysis of the current situation of clinical and scientific young talents

Part A: General Questions for all professions:

*Mandatory Questions

1) How old are you?*

- Younger than 20 years
- 21 – 25 years
- 26 - 30 years
- 31 – 35 years
- 36 – 40 years
- Over 40 years old

2) What gender do you identify as?*

- Male
- Female
- Diverse
- I'd rather not say

3) What discipline are you working in?*

- Medical field
- Biology
- Physics

4) When did you first find out about Radiation Sciences as a potential work field?*

- During University, e.g. during lectures
- Through an advertised PhD thesis

- Through private contacts (e.g. Friends, family)
- Through (free text answer)

5) Do you see your long-term future in the field of radiation research or radiation therapy?*

- Yes, I am sure.
- Probably.
- Undecided
- Rather not
- No, I do not.

6) Would you like to comment on your previous reply?

(Free Text)

7) In your opinion, what "image" does radiation research have?*

- Positive
- Rather positive
- Neutral
- Rather negative
- Negative

8) Would you like to comment on your previous reply?

(Free Text)

9) Are you a member of a professional society in the field of radiation research or radiation therapy?*

- Yes, I am.
- Yes, in more than one.
- I am not, because (free answer)

10) Do you feel adequately represented in your society (e.g. DEGRO, DeGBS, DGMP)?

- Yes, I do.
- No, I do not.

11) Would you like to comment on your previous reply?

(Free Text)

12) Are you actively involved in teaching and/or supervision?*

- Yes, I am involved in both.
- Yes, I am involved in teaching.
- Yes, I am supervising doctoral students.
- No, I am not.
- I am not, but I would like to be involved.

13) Are you actively involved in committee work?*

- Yes, I am.
- No, but I would like to be involved.
- No, I am not and I do not want to at the moment.

Part B: Occupational field specific questions

You will only see questions that are specific to your field of work. The respective assignment is a result from your answer to the question, which field you are working in.

Medical professionals questionnaire

14) What is your current educational status?*

- Medical student
- Assistant Physician
- Medical Specialist
- Senior Physician

15) What is your highest educational degree?*

- In university.
- I have finished my medical studies.
- Doctoral degree.
- Habilitation

16) Where do you see your long-term future in radiation therapy?*

- University hospital
- Private practice
- Center of maximal care
- I do not see myself working in Radiation Therapy, because (free answer)

17) Do you have sufficient support for your research projects?*

- Yes, I have.
- No, I do not
- I have no research projects.

18) Would you like to comment on your previous reply?

(Free text)

19) If you have a research project: Do you carry out your research work within regular working hours?

- Yes, always.
- Yes, most of the time.
- No.
- Other option (free text)

20) If you work in research: How much time do you invest in research work OUTSIDE your working hours per week?

- Less than 10h per week
- 10 – 15h
- 15 – 20h
- More than 20h per week

21) How do you rate the quality of your education?*

Very low (0) – Very well (100)

22) How would you optimize your education?

(Free text)

23) If you had to choose today, would you opt for radiation therapy as your field of expertise again? *

- Yes, I would.
- No, I would not.

24) Does your employer provide sufficient support regarding your Career goals?*

- Yes.
- No.

- I have not spoken about career goals with my employer.

25) How do you rate your subjective workload in the CLINIC?*

Low (0) – High (100)

26) How do you rate your subjective workload in RESEARCH?

Low (0) – High (100)

27) To what percentage are you employed?*

(Free text answer)

28) How many hours per week do you usually work in total?*

(Free text answer)

29) Is your contract*

- Temporary?
- Permanent?
- How long is your temporary contract? (free text)

30) Do you have children or relatives to be cared for?*

- Yes, I do.
- No, I do not.
- I'd rather not say.

31) If you have children or dependents to care for:

- Is there a stand-in arrangement so that urgent activities can be taken over in the event of illness?
- If your child/relative is ill, do you stay at home?
- Do you make up for lost working hours in the evenings?
- Is there a possibility of part-time employment?

Options for all: Yes/Sometimes/No

32) Where do you see the biggest problems in planning a long-term career radiotherapy?*

- Lack of perspective for the future
- Lack of compatibility of career and job

- Work Life Balance
- Lack of further educational possibilities.
- Altered working environment through artificial intelligence
- I do not see a problem in planning a long-time Career in radiation therapy.
- Economic pressure.
- Lack of equality.
- Other (Free text)

33) Do you have additional comments to this question?

(Free text)

34) How well would you rate the possibility of interdisciplinary and translational re-search at your location?*

Low (0) – High (100)

35) What kind of support would you like to receive from your society?

(Free text field)

Questionnaire Biology

36) What is your current educational status?*

- Student
- PhD Student
- PostDoc
- Junior Group Leader
- Group Leader

37) What is your highest university degree?*

- Bachelor/Master
- PhD
- Habilitation
- Professorship

38) How long are you working in research?*

- Less than 5 years
- 5 to 10 years
- More than 10 years

39) Is your contract *

- Temporary?
- Permanent?
- How long is your temporary contract? (Free text reply)

40) Would you like to tell us a little more about it? (e.g. how long is your contract, how many temporary contracts did you have? How long until you had a permanent contract?)

(Free text)

41) Are your tasks limited to the contractually agreed job description?*

- Yes, they are
- No, they are not.
- I'd rather not say.

42) Do you carry out your research within regular working hours or outside of your laboratory obligations?*

- Yes, always.
- Yes, most of the time.
- No, I only do research in my spare time.
- Other option (free text)

43) Would you like to comment on your previous reply?

(Free text)

44) Do you have children or relatives to be cared for?*

- Yes, I do.
- No, I do not.
- I'd rather not say.

45) If you have children or dependents to care for:

- Is there a stand-in arrangement so that urgent activities can be taken over in the event of illness?
- If your child/relative is ill, do you stay at home?
- Do you make up for lost working hours in the evenings?
- Is there a possibility of part-time employment?

For all answers: Yes/Sometimes/No

46) Where do you see yourself in the future?*

- University Research
- Industry
- Neither (Free text reply)

47) If you do NOT want to stay in university research, why is that?

- Lack of future perspectives
- Lack of support through employer/supervisor.
- Lack of compatibility of work and family planning
- Work Life Balance
- Uncertain contract terms
- I would like to stay, but I cannot (e.g. lack of funding or similar)
- Other reasons

48) If you do want to stay in university research, what are your reasons?

(Free text reply)

49) Do you see your future in radiation research?*

- Yes, I do.
- No, I do not.

50) Would you like to comment on your previous reply?

(Free text)

51) Does your employer provide sufficient support regarding your Career goals?*

- Yes
- No
- I have not spoken about Career goals with my employer.

52) Would you choose a career in biological radiation research again?*

- Yes, I would.
- No, I would not.
- Unsure

53) How well would you rate your Career options in radiation biology? *

Low (0) – High (100)

54) Do you rate the career opportunities in radiation biology better or worse than in other biological research fields?*

- Better
- Worse
- I do not know.

55) How do you rate your subjective workload?*

Low (0) – High (100)

56) To what percentage are you employed? *

(Free text reply)

57) How many hours per week do you usually work in total?

(Free text reply)

58) How well would you rate the possibility of interdisciplinary and translational re-search at your location?*

Low (0) – High (100)

59) What kind of support would you like to receive from your society?

(Free text)

60) What kind of support would you like from politics (e.g. WissZeitVG, funding, visibility)?

(Free text)

Questionnaire Physics

61) What is your current educational status?*

- Student
- Doctoral student
- PostDoc
- Junior group leader
- Group leader
- Currently working on my MPE.
- MPE in medical radiation protection.

62) What is your highest university degree?*

- I am still a student
- Bachelor
- Master
- PhD
- Habilitation
- Professorship

63) Where do you see your long-term future?*

- University hospital
- Private practice
- Center of maximal care
- In industry related to radiation therapy.
- Research Institute outside of university
- I do not see myself working in Radiation Therapy, because (free text reply)

64) If you do not see your future in university research, why is that?*

- Lack of perspective for the future
- Lack of compatibility of career and job
- Work Life Balance
- Lack of further educational possibilities.
- Altered working environment through artificial intelligence
- I do not see a problem in planning a long-time career in radiation therapy.

65) If you do want to stay in university research, what are your reasons?

(Free text)

66) Do you have sufficient support for your research projects?*

- Yes, I have.
- No, I do not
- I have no research projects.

67) Would you like to comment on your previous reply?

(Free text)

68) If you have a research project: Do you carry out your research work within regular working hours?

- Yes, always.
- Yes, most of the time.
- No, I only do research in my spare time.
- Other option

69) If you work in research: How much time do you invest in research work OUTSIDE your working hours per week?

- Less than 10h per week
- 10-15h
- 15-20h
- More than 20h per week

70) If you had to choose today, would you opt for radiation therapy as your field of expertise again?*

- Yes, I would.
- No, I would not.

71) Would you like to comment on that?

(Free text)

72) Does your employer provide sufficient support regarding your Career goals?*

- Yes
- No
- I have not spoken about Career goals with my employer.

Please answer the following two questions only if they are also doing clinical or research work respectively. For example, if you do not do research, please leave the question unanswered. Thank you very much!

73) In case you are working in the clinic: How do you rate your subjective workload in the clinic?

Low (0) – High (100)

74) In case you are also working in research: How do you rate your subjective workload in research?

Low (0) – High (100)

75) Is your contract*

- Temporary?
- Permanent?
- How long is your temporary contract? (free text reply)

76) Do you have children or relatives to be cared for?*

- Yes, I do.
- No, I do not.
- I'd rather not say.

77) If you have children or dependents to care for:

- Is there a stand-in arrangement so that urgent activities can be taken over in the event of illness?
- If your child/relative is ill, do you stay at home?
- Do you make up for lost working hours in the evenings?
- Is there a possibility of part-time employment?

For all: Yes/Sometimes/No

78) Where do you see the biggest problems in planning a long-term career radiotherapy?*

- Lack of perspective for the future
- Lack of compatibility of career and job
- Work Life Balance
- Lack of further educational possibilities.
- Altered working environment through artificial intelligence
- I do not see a problem in planning a long-time Career in radiation therapy.
- Economic pressure.
- Lack of equality
- Other

79) How well would you rate the possibility of interdisciplinary and translational re-search at your location?*

Low (0) – High (100)

80) What kind of support would you like to receive from your society?

(Free text)
