# Supplementary material for: German radiation oncology’s next generation: a web-based survey of young biologists, medical physicists, and physicians—from problems to solutions
Source: Strahlenther Onkol. 2024 Oct 22;200(12):1005–24. doi: 10.1007/s00066-024-02305-8 (PMC11588816; doi:10.1007/s00066-024-02305-8)
Supplement: Supplementary file 2 — Supplementary Material 2 Table 1. Characteristics of survey participants (n = 218). Absolute numbers are given in brackets. Numbers may not add up to 100% due to rounding error or missing values. Abbreviations: d diverse; f female; m male; n/a not applicable. [file 66_2024_2305_MOESM2_ESM.pdf]

Strahlentherapie und Onkologie

German Radiation Oncology's Next Generation: A Web-Based Survey of Young Biologists, Medical Physicists, and Physicians—From Problems to Solutions

Thomas Weissmann [1,2,3,†], Lisa Deloch [1,2,3,4,†,*], Maximilian Grohmann [3,5], Maike Trommer [3,6,7], Alexander Fabian [3,8], Felix Ehret [3,9], Sarah Stefanowicz [3,10], Alexander Rühle [3,11,12], Sebastian Lettmaier [1,2], Florian Putz [1,2], Maya Shariff [1,2], Simone Wegen [3,13], Johann Matschke [3,14, 15], Elena Sperk [16,†] and Annemarie Schröder [3,17,18,†]

† contributed equally

* Corresponding author: Dr. Lisa Deloch, Radiation Osteoimmunology at Translational Radiobiology, Department of Radiation Oncology, Uniklinikum Erlangen, Germany; lisa.deloch@uk-erlangen.de;

Supplementary Material 2

Table 1. Characteristics of survey participants (n=218). Absolute numbers are given in brackets. Numbers may not add up to 100% due to rounding error or missing values. Abbreviations: d, diverse; f, female; m, male; n/a, not applicable.

	Physicians	Biologists	Physicists	All
Participants	51% (89)	27% (59)	32% (70)	100% (218)
Age				
<20 years	1% (1)	0% (0)	0% (0)	1% (1)
21-25 years	7% (6)	7% (4)	23% (16)	12% (26)
26-30 years	30% (27)	34% (20)	27% (19)	30% (66)
31-35 years	42% (37)	17% (10)	20% (14)	28% (61)
36-40 years	10% (9)	22% (13)	20% (14	17% (36)
>40 years	10% (9)	20% (12)	10% (7)	13% (28)
Sex				
f:m:d	42%:56%:0% (37:50:0)	73%:27%:0% (43:16:0)	43%:53%:1% (30:37:1)	51%:47%:1% (110:103:1)
Highest academic degree				
Student	9% (8)	0% (0)	6% (4)	6% (12)
Graduate[1]	34% (30)	46% (27)	67% (47)	48% (104)
Dr.med. (MD)	49% (44)	n/a	n/a	20% (44)
Dr.rer.nat	n/a	42% (25)	26% (18)	20% (43)
Habilitation	8% (7)	8% (5)	0% (0)	6% (12)
Associate Professor	0% (0)	3% (2)	1% (1)	1% (3)
Contract				
Fixed-term	76% (68)	75% (44)	56% (39)	69% (151)
permanent	24% (21)	25% (15)	44% (31)	31% (67)
Member of a professional society				
yes:no	87%:13% (77:12)	66%:37% (39:20)	54%:46% (38:32)	71%:29% (154:64)

[1] Includes graduated physicians without doctoral thesis.
